# National Trends in Statin Use in Lithuania from 2010 to 2021

**DOI:** 10.3390/medicina59010037

**Published:** 2022-12-24

**Authors:** Gytis Makarevičius, Egidija Rinkūnienė, Jolita Badarienė

**Affiliations:** 1 Centre for Neurology, Vilnius University, LT-03101 Vilnius, Lithuania; 2 Clinic of Cardiac and Vascular Diseases, Medical Faculty, Vilnius University, LT-03101 Vilnius, Lithuania

**Keywords:** statins, use trends, cardiovascular diseases, prevention, hypercholesterolemia

## Abstract

*Objective:* In Lithuania, no comprehensive national research on statin utilization and trends has yet been undertaken. Nonetheless, this knowledge is critical for the healthcare system to identify key areas for development. We aimed to analyze trends in statin utilization in Lithuania from the past 12 years considering changes in reimbursement policies and the publication of updated international CVD prevention guidelines. *Methods:* We performed a retrospective, descriptive study of statin utilization in Lithuania from 2010 to 2021. The data were obtained from PharmaZOOM LT, an independent software supplier with nationwide coverage on pharmaceutical market data. The data coverage was 95%. We used anatomical therapeutic chemical (ATC) classification for data extraction and calculated defined daily doses (DDDs) according to the ATC/DDD Toolkit of World Health Organization according to statin dose in a pill. *Results:* Statin use increased overall from 8.28 DDD/TID in 2010 to 96.06 DDD/TID in 2021. The annual growth rate in sales of statin DDD/TID was 22.28%. The increase was mostly due to the increase in moderate- and high-intensity statins. The increases coincided with changes in reimbursement policy or the publication of international guidelines. Polypill use in Lithuania began steadily increasing after 2016 and reached 19.37% of the total DDD/TID of statins in 2021. *Conclusions:* The use of statins has increased dramatically in Lithuania over the last decade. Changes in statin reimbursement regulations in the country, as well as worldwide cardiovascular preventive recommendations aiming at lower LDL-C objectives, fueled the progress.

## 1. Introduction

Until 1976, when the first HMG-CoA reductase inhibitor (statin), compactin, was discovered by Akira Endo, there was no highly effective treatment of hypercholesterolemia (although different options, such as nicotinic acid, resins and fibrates, had been tried) [1]. Compactin was never marketized, but its success led to commercial statins (lovastatin, simvastatin, pravastatin, fluvastatin, atorvastatin and rosuvastatin) being discovered not long afterwards [1]. Later clinical trials, such as the Scandinavian Simvastatin Survival Study (known as the 4S study) in 1994, proved statins to be effective in the secondary prevention of coronary events [2]. Statin therapy for primary prevention has also been proven beneficial [3,4,5,6,7]. In addition, recent studies indicate that statin use is also associated with a reduced risk of carotid restenosis after endarterectomy, cardiovascular (CVD) and even non-CVD mortality [8,9]. However, the benefit seems to be more pronounced in patients in higher-risk groups and/or with higher baseline LDL-C levels; thus, the debate about whether patients with lower baseline LDL-C and/or patients in low- or medium-risk groups should be treated is ongoing [3,4,5,6,7]. Nevertheless, the existing evidence has convinced guideline committees in Europe and the world. Since the first recommendations on the initiation of statin therapy for primary and secondary prevention were first proposed by the Second Joint Task Force of European and other Societies on Coronary Prevention in 1998, the European Atherosclerosis Society (EAS) and other medical societies have worked on lowering the threshold of the LDL-C goal for certain patient risk groups [10,11,12]. As a result, the population for whom statin therapy might be indicated has expanded, and this has resulted in a continuous increase in statin consumption worldwide [13]. Compared with other Western European countries, Lithuania’s statin consumption grew slowly. Strict statin reimbursement policies might be to blame, as until 2008, statin therapy was only reimbursed for secondary prevention and only for 6 months. In 2006, to improve statin prescription, the Lithuanian High Cardiovascular Risk (LitHiR) primary prevention program was launched [14]. The program, together with the implementation of more liberal statin reimbursement policies in 2009, 2015 and 2019 and the publication of new international CVD guidelines calling for stricter LDL-C targets, could be responsible for the sharp increase in statin consumption. Detailed nationwide studies on statin utilization and its trends have never been conducted in Lithuania. Nevertheless, this knowledge is crucial for the national healthcare system to identify major points for improvement, as well as understand the effects of the implemented policies on a medical market. Therefore, we analyzed the trends in statin utilization in Lithuania from the past 12 years by considering new changes in reimbursement policies and the publication of updated international CVD prevention guidelines. 

## 2. Materials and Methods

We performed a retrospective, descriptive study of statin utilization in Lithuania from 2010 to 2021. The data were obtained from the PharmaZOOM LT database. PharmaZOOM is a software created by SoftDent that enables access to data from the Lithuanian medicine market. PharmaZOOM LT collects and systemizes weekly sell-in data of medicinal substances, medical devices and dietary supplements sold to Lithuanian pharmacies, hospitals and other institutions by the leading wholesale companies in Lithuania. The wholesale data coverage of the database was approximately 95% of all sales in Lithuania. The data on statin utilization from 2010 to 2021 were extracted using anatomical therapeutic chemical (ATC) classification codes [15]. The following codes were used: “C10AA—HMG CoA reductase inhibitors”, “C10BA Combinations of various lipid modifying agents” and “C10BX Lipid modifying agents in combination with other drugs”. The extracted data consisted of package information, substance, strength, brand, product name, producer, corporation, dispensing type, product type (generic, branded generic and patent), quantity of packages, units, defined daily doses (DDDs) sold and year of the sale. DDD was calculated according to the ATC/DDD Toolkit of the World Health Organization (WHO) [15]. Since the WHO have not defined a number of units per DDD for combination drugs (ATC codes C10BA and C10BX) and DDD, for these substances, they are based only on dosing frequency (whereas there is a different dose of statin in a certain pill). Moreover, since we sought to evaluate the trends in statin utilization, we defined the DDD of combination medicine according to the dose of statin in the medication. There are two types of combinations in the Lithuanian medicine market: combinations with atorvastatin and combinations with rosuvastatin. Thus, for combination medicine, 1 DDD was defined as 20 mg of atorvastatin if it was a combination with atorvastatin and 10 mg of rosuvastatin if it was a combination with rosuvastatin [15]. We used the data to calculate the DDD for a thousand inhabitants per day (TID). The data on the inhabitant count in Lithuania each year were extracted from the official website of the Department of Statistics of Lithuania. We also grouped statins into three groups according to therapy intensity (as they are classified by the American College of Cardiology (ACC) and the American Heart Association (AHA)) [16]. Accordingly, sales of ≥40 mg of atorvastatin and ≥20 mg of rosuvastatin were defined as high-intensity statin therapy; 10–30 mg of atorvastatin, 5–15 mg of rosuvastatin, 80 mg of fluvastatin and 20 mg of simvastatin as moderate-intensity; and 10 mg of simvastatin as low-intensity statin therapy [16]. The sales of other forms of statins (pitavastatin, lovastatin and pravastatin) and other strength groups not mentioned in the description were not recorded because those statins are not available/registered in Lithuania. All analyses were performed using Microsoft Excel version 2112.

## 3. Results

**Figure 1:**
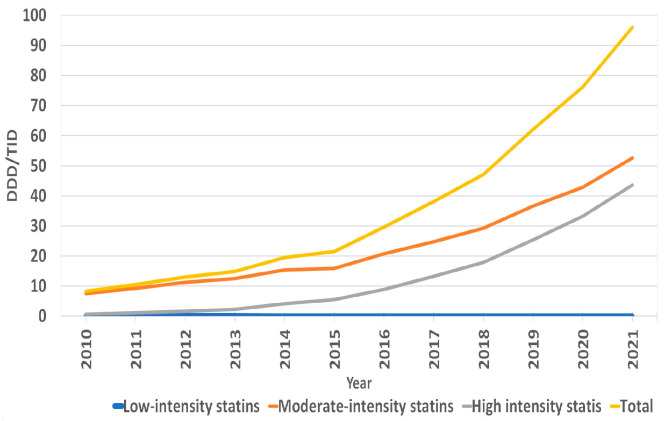
Annual sales of statins in DDD/TID according to therapy intensity.

**Figure 2:**
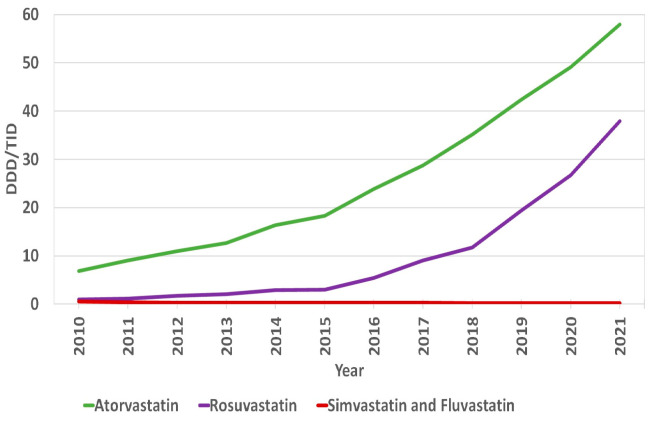
Annual sales of statins in DDD/TID according to types of statins.

**Figure 3:**
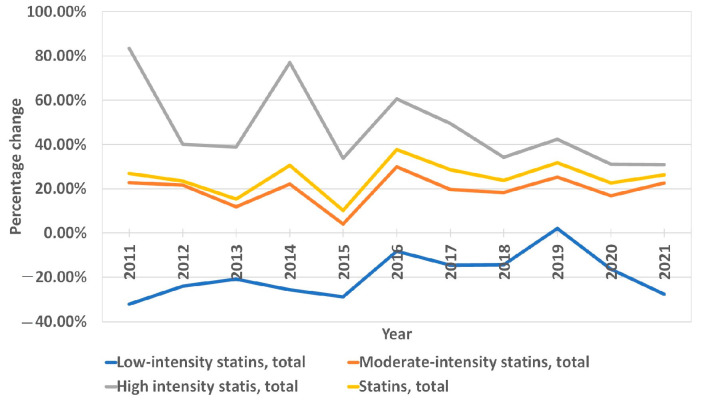
Annual percentage change in statin sales according to therapy intensity.

**Figure 4:**
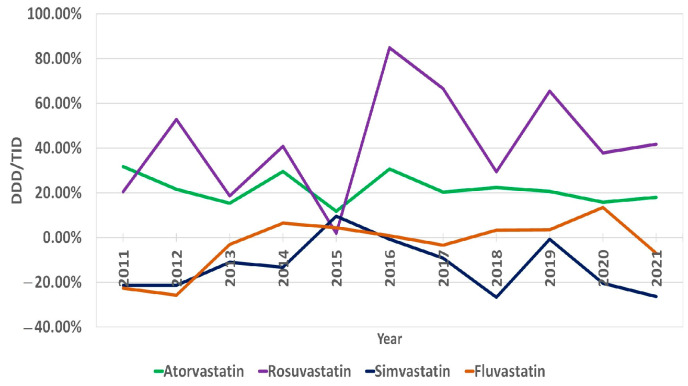
Annual percentage change in statin sales according to types of statins.

The overall use of statins in Lithuania increased by 1060%, from 8.28 DDD/TID in 2010 to 96.06 DDD/TID in 2021 (Table S1). The increase was mostly due to the increase in moderate- and high-intensity statins. The utilization of the latter increased from 0.66 DDD/TID in 2010 to 43.53 in 2021, and the former increased from 7.52 DDD/TID in 2010 to 52.52 in 2021. The use of low-intensity statins decreased from 0.11 DDD/TID in 2010 to 0.01 in 2021 (Figure 1, Table S1). In 2021, the moderate-intensity statins accounted for the majority of total statin DDD/TID (54.67%), while high- and low-intensity statins accounted for 45.32% and 0.01%, respectively. Atorvastatin sales increased from 6.84 DDD/TID in 2010 (82.61% of total statin DDD/TID) to 57.95 in 2021, while the total share of statin DDD/TID decreased to 60.33%. This was due to an increase in rosuvastatin sales from 0.94 to 37.91 DDD/TID (11.35% and 39.46% of total statin DDD/TID, respectively) over the period (Figure 2, Table S1). On the contrary, the use of simvastatin and fluvastatin decreased from 0.27 and 0.23 (3.26% and 2.78% of total statin DDD/TID) in 2010 to 0.05 and 0.16 DDD/TID (0.05% and 0.17% of total statin DDD/TID) in 2021, respectively. From 2010 to 2021, statin utilization in Lithuania increased annually, without any periods of decrease (Figure 3 and Figure 4, Table S1).

**Figure 5:**
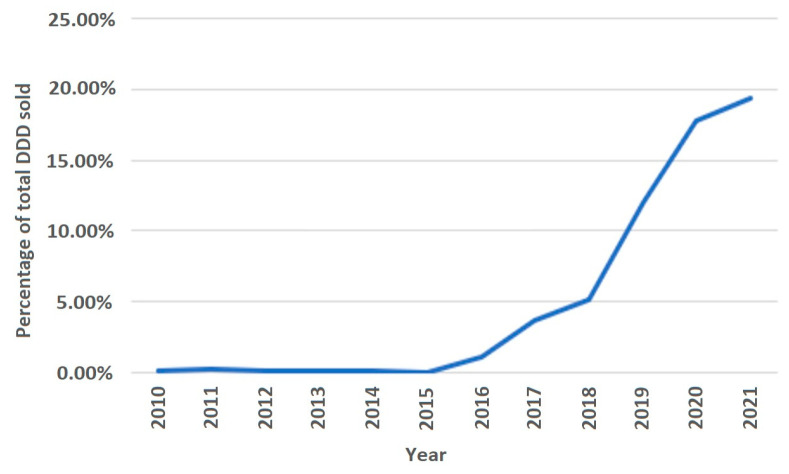
Statin sales in polypills of total statin sales.

**Figure 6:**
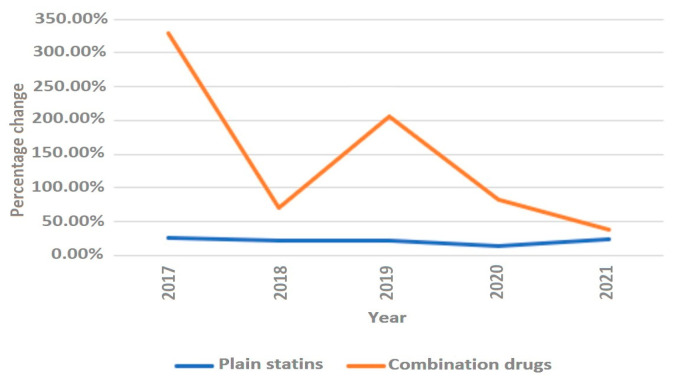
Annual rate of change in polypills and plain statins.

The average annual growth rate (percentage change for the following year vs. the previous year) in sales of statin DDD/TID was 22.28%. The years 2011, 2014, 2016 and 2019 were marked by the largest annual increases in statin prescription, with values of 26.92%, 30.55%, 37.69% and 31.67%, respectively. The increases in 2011 and 2014 were mainly driven by a significant increase in high-intensity statin sales (increases of 83.47% and 76.90% from last year, respectively). The increases in 2016 and 2019 were likely caused by more even increases in both moderate- and high-intensity statins (in 2016, moderate-intensity statin sales increased by 29.83%, while high-intensity statin sales increased by 60.50%). Similarly, in 2019, the increases were 25.22% and 42.29%. The smallest annual increase was seen in 2015 (10.13%). The most considerable average annual growth rate was seen for rosuvastatin (33.59%) and in the high-intensity statin group (38.12%). As for statins in combination with other drugs (polypills), the share of total statin DDD/TID began steadily increasing after 2016 (Figure 5, Table S1). As of 2021, 19.37% of the total DDD/TID of statins was sold as a polypill. The DDD/TID of combination drugs increased from 0.01 to 18.60 in 2021, whereas plain statin therapy increased from 8.27 to 77.46 DDD/TID (with 68.10% and 20.33% average annual growth rates, respectively) (Figure 6, Table S1). In 2021, combinations with other lipid-lowering agents (ezetimibe) comprised only a small percent of total combination medication DDD/TID (0.21 DDD/TID or 1.13%), whereas combinations with other drugs, namely, antihypertensives and diuretics (amlodipine, perindopril, valsartan and indapamide), comprised the majority (18.39 DDD/TID or 98.87%).

**Figure 7:**
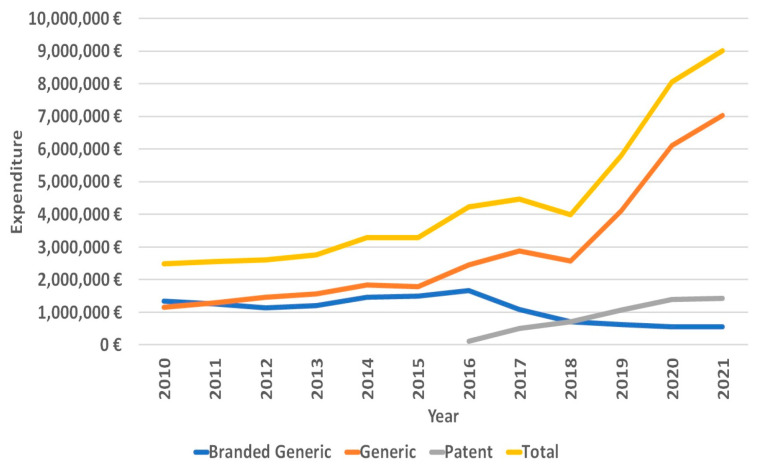
Expenditure on statins.

As for the expenditure on statins, over the period between 2010 and 2021, the total expenditure on statins in Lithuania grew by about 260% from EUR 2,490,987 in 2010 to EUR 9,013,100 in 2021 (Figure 7). However, the mean expenditures for DDD decreased by 66.67% from EUR 0.27 for DDD in 2010 to EUR 0.09 for DDD in 2021.

## 4. Discussion

Our study found that statin sales in Lithuania increased over the period of 12 years from 8.28 in 2010 to 96.06 DDD/TID in 2021. Similarly, for the past decade, an increase in lipid-modifying agents has been seen globally [5,13,17,18,19,20]. Europe and North America have seen the greatest increase [13]. By contrast, Lithuania consumed a small amount of lipid-lowering medication for a relatively long period of time. Until 2015, less than 20 DDD/TID of statins were sold daily, while similar sale results were achieved a decade earlier in many Western and Northern European countries [5,21]. Thus, the 96.06 DDD/TID in 2021 could be considered an improvement. However, there is still room for improvement, taking into account Western and Northern European countries, such as Norway, a country that has one of the lowest cardiovascular mortalities in Europe and a statin consumption of 148 DDD/TID in 2020 [22]. Despite recent improvements in statin consumption in Europe, there still seems to be a gap between guideline recommendations and real-life clinical practices, with most of the high-risk patients not meeting the LDL-C levels recommended by the ESC/EAS [23,24]. The improvements in the clinical management of patients will likely result in a further increase in statin consumption in Europe, notably high-intensity statin therapy, as the suboptimal utilization of high-intensity statins is likely one of reasons for the non-achievement of the recommended LDL-C levels [25]. 

We recorded that rosuvastatin and atorvastatin had the highest sales over the studied period, with the latter being the most widely used. Nevertheless, after 2015, the rosuvastatin annual percentage change rates exceeded those of atorvastatin. If this tendency persists, rosuvastatin sales are likely to outstrip atorvastatin sales in the future. One of the reasons for this trend could be the increased sales of combination medication that in the majority of combination choices are with rosuvastatin. Similarly, in other countries, atorvastatin and rosuvastatin are also frequently used; however, unlike in Lithuania, the use of simvastatin is also prevalent [17,18,20,26]. The observed “popularity” of atorvastatin and rosuvastatin and the “unpopularity” of simvastatin and fluvastatin could be explained by a number reasons, including atorvastatin’s demonstrated superiority in secondary prevention [6], the high efficacy of rosuvastatin in reducing LDL-C levels [27] and its availability for use in different combinations with other cardiological medicines as polypills in the market, the more successful marketing strategies of atorvastatin and rosuvastatin companies and the potency of high doses of rosuvastatin and atorvastatin for achieving >50% LDL-C reduction (high-intensity statin therapy was marked by the largest average annual growth rate in Lithuania) [16]. 

Interestingly, we also recorded significant increase in combination medication sales, with an average annual growth rate of about 68.10%. In 2021, statins in polypills accounted for about 19% of total sales. The trend could have been influenced by the recent studies showing that treatment with polypills as opposed to multiple pills results in better adherence as well as by the successful marketing strategies of said marketing companies [28]. 

**Figure 8:**
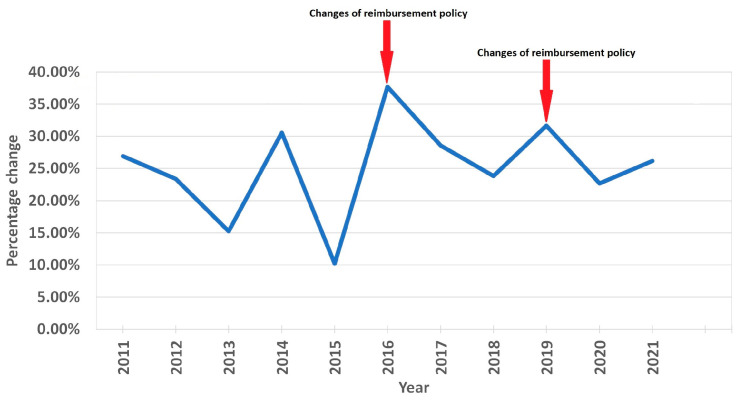
Annual percentage change in statin sales and changes in national reimbursement policies.

Strict statin reimbursement policies might be one of the key reasons why the prescription frequency of statins was low in Lithuania until 2015, as from 2008 to 2015 statins were reimbursed only for secondary prevention and for patients in a high CVD risk group with total TC ≥ 7.5 mmol/L, LDL-C ≥ 6.0 mmol/L, or TG ≥ 4.5 mmol/L. Patients for whom statin therapy is not reimbursed might be less willing to adhere to treatment even if the doctor prescribed the treatment. As a recent study in Canada has shown, high out-of-pocket spending is one of the factors predicting nonadherence [29]. In addition, from 2000 until December 2015, the right to prescribe statins was reserved only for cardiology specialists, complicating the process of statin prescription. To reinforce the claim that statin prescription is heavily influenced by reimbursement regulations, it is worth mentioning that the two most potent increases in statin prescription occurred directly after new reimbursement policies had been implemented (large increases in 2016 and 2019) (Figure 8). In December 2015, 80% reimbursement became available for patients in a high-risk group whose blood LDL-C was ≥3.0 mmol/L and for patients in a very high-risk group if LDL-C was ≥1.8 mmol/L. In addition, general practitioners and neurologists (in the presence of cerebrovascular disease) acquired the right to prescribe reimbursed statins. In 2019, 100% reimbursement for statins was implemented for very high-risk (LDL-C ≥ 1.8 mmol/L), high-risk (LDL-C ≥ 3.0 mmol/L) and intermediate-risk (LDL-C ≥ 3.0 mmol/L) groups, as well as for people with a family history of premature coronary artery disease (LDL-C ≥ 5.0 mmol/L). After more liberal statin prescription policies were implemented in December 2015, the statin DDD/TID increased for about 76 DDD/TID in over 6 years as opposed to an increase of about 12 DDD/TID in over 6 years of pre-reimbursement. Thus, public authorities, through the assumption of responsibilities and the reimbursement of treatments, play an equally significant role in improving healthcare for patients.

In addition to new reimbursement policies, updated CVD prevention guidelines might have also had a positive effect on statin utilization in Lithuania, especially the utilization of high-intensity statins. New updated ESC CVD guidelines were published in 2011 [30], 2016 [11], 2019 [11] and 2021 [12]. The publication of updated guidelines seems to be a factor for stimulating statin use, as major increases in statin utilization were recorded that same year after the updated guidelines had been published (Figure 7) (although the increases in 2016 and 2019 also coincided with reimbursement policy changes). However, the connection is not as clear as it is with reimbursement changes (the 2014 increase could not be explained by a release of ESC CVD guidelines). The impact of these guidelines could be seen though changes in prescribed statin therapy intensity (we recorded a decreased use of low-intensity statins, and an increased use of moderate- and high-intensity stains, especially the latter), as the guidelines called for stricter LDL-C goals and encouraged the use of high-intensity statins to achieve those goals [11,12,30,31].

Despite recent improvements in preventive cardiology, CVD mortality in Lithuania is not yet declining [21]. Lithuania remains a country with a very high CVD risk. Thus, it is safe to say that statin utilization in Lithuania is far from “optimal” [12]. To achieve better CVD mortality results, there is likely a need to push statin consumption even farther behind the achieved amount of 96.06 DDD/TID. The “optimal” amount, considering the high prevalence of ASCVD, could be even higher at this point than that of Western or Northern European countries. However, as the highly successful The North Karelia Project showed and as Vancheri et al. stressed, educational programs that move society towards a healthier lifestyle are equally important [5,32]. This is also emphasized in the EHI 2016 report, which mentions some additional areas for Lithuania to improve. These include the prevalence of obesity, fruit and vegetable consumption, tobacco and alcohol consumption, the prevalence of raised blood pressure, etc. [33]. Improvements in these factors would likely result in an improvement in CVD mortality. To reach better CVD mortality and morbidity results in Lithuania, an improvement in statin consumption (the promotion of public cholesterol screening programs, educational programs for doctors to ensure clinical guideline adherence, etc.) and the implementation of socioeconomical programs (tobacco, alcohol, sugar taxes, smoke-free laws, educational programs in schools, dietary recommendations, etc.) that promote healthier lifestyles are essential.

## 5. Conclusions

Statin utilization in Lithuania has improved over the past decade. A number of factors have contributed to the improvement, including changes in statin reimbursement policies in the country and international cardiovascular prevention guidelines. Higher statin sales could also indicate that an increasing number of Lithuanian doctors adapt strict European guideline policies in their everyday clinical practices. However, CVD mortality remains high in Lithuania. Further improvement in statin utilization and the implementation of socioeconomical measures should both be applied to achieve better CVD morbidity and mortality results. 

## Data Availability

Restrictions apply to the availability of these data. The data were obtained from the PharmaZOOM LT database of SoftDent and are available from the authors with the permission of SoftDent.

## Supplementary Materials

The following supporting information can be downloaded at: https://www.mdpi.com/article/10.3390/medicina59010037/s1, Table S1: Statin sales in Lithuania from 2010 to 2021.
