# Supplementary material for: National Trends in Statin Use in Lithuania from 2010 to 2021
Source: Medicina (Kaunas). 2022 Dec 24;59(1):37. doi: 10.3390/medicina59010037 (PMC9864999; doi:10.3390/medicina59010037)
Supplement: Supplementary file 1 [file medicina-59-00037-s001.zip › medicina-2010617-supplementary.pdf]

**Supplementary Table S1.** Statin sales in Lithuania from 2010 to 2021.

| **Year** | 2010 | 2011 | 2012 | 2013 | 2014 | 2015 | 2016 | 2017 | 2018 | 2019 | 2020 | 2021 |
|---|---|---|---|---|---|---|---|---|---|---|---|---|
| Inhabitant count | 3102741 | 3025027 | 2987523 | 2956558 | 2931612 | 2903951 | 2870351 | 2824030 | 2801501 | 2792209 | 2796025 | 2800990 |
| **Statin sales, DDD** | | | | | | | | | | | | |
| Atorvastatin, total | 7748790 | 9946153.5 | 11948124.5 | 13631766.25 | 17516972 | 19384898 | 25032788.15 | 29625543.5 | 35959679.5 | 43225164.15 | 50149703 | 59241311.2 |
| Atorvastatin, plain | 7737030 | 9910026 | 11922497 | 13606243.75 | 17494554.5 | 19368733 | 24685853.15 | 28159373.5 | 33533534.5 | 37596084.15 | 40710743 | 46921301.2 |
| Atorvastatin, in combinations | 11760 | 36127.5 | 25627.5 | 25522.5 | 22417.5 | 16165 | 346935 | 1466170 | 2426145 | 5629080 | 9438960 | 9438960 |
| Rosuvastatin, total | 1066046 | 1252418 | 1889173 | 2216300.003 | 3094823 | 3124532 | 5708445 | 9354522 | 12002391 | 19787282.8 | 27297414.46 | 38758269.15 |
| Rosuvastatin, plain | 1066046 | 1252418 | 1889173 | 2216300.003 | 3094823 | 3124532 | 5708445 | 9354522 | 11947221 | 17845612.8 | 22961484.46 | 32059978.95 |
| Rosuvastatin, in combinations | 0 | 0 | 0 | 0 | 0 | 0 | 0 | 0 | 55170 | 1941670 | 4335930 | 6698290.2 |
| Simvastatin, total | 307170 | 214523.3333 | 166654 | 146623.3333 | 126201.3333 | 137051 | 134466.6667 | 120216.6667 | 87473.33333 | 86480 | 68890 | 50800 |
| Fluvastatin, total | 259168 | 195701.3333 | 143434.6667 | 137610.6667 | 145142.6667 | 150061.3333 | 149594.6667 | 142202.6667 | 145712 | 150266.6667 | 170613.3333 | 159040 |
| Low-intensity statins, total | 119844 | 79404.66667 | 59611.33333 | 46667.33333 | 34416 | 24290.99999 | 22040 | 18530 | 15746.66667 | 16020 | 13410 | 9720 |
| Moderate-intensity statins, total | 8517030 | 10198019.5 | 12247946.83 | 13556650.92 | 16412787 | 16899483.33 | 21686782.48 | 25523280.83 | 29936133.17 | 37360498.82 | 43708224.79 | 53693746.35 |
| High intensity statis, total | 744300 | 1331372 | 1839828 | 2528982.004 | 4435936 | 5872768 | 9316472 | 13700674 | 18243376 | 25872674.8 | 33964986 | 44505954 |
| Total of C10AA | 9369414 | 11572668.67 | 14121758.67 | 16106777.75 | 20860721.5 | 22780377.33 | 30678359.48 | 37776314.83 | 45713940.83 | 55678443.62 | 63911730.79 | 79191120.15 |
| Total of C10BA* | 0 | 0 | 0 | 0 | 0 | 0 | 0 | 0 | 0 | 0 | 43290 | 213450 |
| Total of C10BX* | 11760 | 36127.5 | 25627.5 | 25522.5 | 22417.5 | 16165 | 346935 | 466170 | 2481315 | 7570750 | 13731600 | 18804850.2 |
| Total of combination medicine* | 11760 | 36127.5 | 25627.5 | 25522.5 | 22417.5 | 16165 | 346935 | 466170 | 2481315 | 7570750 | 13774890 | 19018300.2 |
| Statins, total | 9381174 | 11608796.17 | 14147386.17 | 16132300.25 | 20883139 | 22796542.33 | 31025294.48 | 39242484.83 | 48195255.83 | 63249193.62 | 77686620.79 | 98209420.35 |

| Statin sales in DDD/ TID and percentage change rate from previous year | | | | | | | | | | | |
|---|---|---|---|---|---|---|---|---|---|---|---|
| Atorvastatin, total | 6.842 | 9.008; 31.66% | 10.957; 21.64% | 12.632; 15.29% | 16.370; 29.59% | 18.289; 11.72% | 23.894; 30.65% | 28.741; 20.29% | 35.167; 22.36% | 42.413; 20.60% | 49.140; 15.86% | 57.946; 17.92% |
| Atorvastatin, plain | 6.832 | 8.975; 31.38% | 10.934; 21.82% | 12.608; 15.32% | 16.349; 29.67% | 18.273; 11.77% | 23.562; 28.94% | 27.319; 15.94% | 32.794; 20.04% | 36.889; 12.49% | 39.891; 8.14% | 45.895; 15.05% |
| Atorvastatin, in combinations | 0.010 | 0.033; 215.10% | 0.024; -28.17% | 0.024; 0% | 0.021; -11.42% | 0.015; -27.20% | 0.331; 2071.33% | 1.422; 329.54% | 2.373; 66.81% | 5.523; 132.79% | 9.249; 67.45% | 12.051; 30.29% |
| Rosuvastatin, total | 0.941 | 1.134; 20.50% | 1.732; 52.74% | 2.054; 18.54% | 2.892; 40.83% | 2.948; 1.92% | 5.449; 84.84% | 9.075; 66.56% | 11.738; 29.34% | 19.415; 65.41% | 26.748; 37.77% | 37.911; 41.73% |
| Rosuvastatin, plain | 0.941 | 1.134; 20.50% | 1.732; 52.74% | 2.054; 18.54% | 2.892; 40.83% | 2.948; 1.92% | 5.449; 84.84% | 9.075; 66.56% | 11.684; 28.74% | 17.510; 49.87% | 22.499; 28.49% | 31.359; 39.38% |
| Rosuvastatin, in combinations | 0 | 0 | 0 | 0 | 0 | 0 | 0 | 0 | 0.054 | 1.905; 3431.14% | 4.249; 123.00% | 6.552; 54.21% |
| Simvastatin, total | 0.271 | 0.194; -21.34% | 0.153; -21.34% | 0.136; -11.10% | 0.118; -13.20% | 0.129; 9.63% | 0.128; -0.74% | 0.117; -9.13% | 0.086; -26.65% | 0.085; -0.81% | 0.068; -20.45% | 0.050; -26.39% |
| Fluvastatin, total | 0.229 | 0.177; -22.55% | 0.132; -25.79% | 0.128; -3.06% | 0.136; 6.37% | 0.142; 4.37% | 0.143; 0.86% | 0.138; -3.38% | 0.142; 3.29% | 0.147; 3.47% | 0.167; 13.39% | 0.156; -6.95% |
| Low-intensity statins, total | 0.106 | 0.072; -32.04% | 0.055; -23.98% | 0.043; -20.89% | 0.032; -25.62% | 0.023; -28.75% | 0.021; -8.20% | 0.018; -14.55% | 0.015; -14.34% | 0.016; 2.07% | 0.013; -16.41% | 0.010; -27.65% |
| Moderate-intensity statins, total | 7.521 | 9.236; 22.81% | 11.232; 21.61% | 12.562; 11.84% | 15.339; 22.10% | 15.944; 3.95% | 20.700; 29.83% | 24.761; 19.62% | 29.276; 18.23% | 36.658; 25.22% | 42.828; 16.83% | 52.519; 22.63% |
| High intensity statis, total | 0.657 | 1.206; 83.47% | 1.687; 39.93% | 2.344; 38.90% | 4.146; 76.90% | 5.541; 33.65% | 8.892; 60.50% | 13.292; 49.47% | 17.841; 34.23% | 25.386; 42.29% | 33.281; 31.10% | 43.533; 30.80% |
| Total of C10AA | 8.273 | 10.481; 26.69% | 12.950; 23.56% | 14.926; 15.25% | 19.495; 30.62% | 21.492; 10.24% | 29.282; 36.25% | 36.649; 25.16% | 44.706; 21.99% | 54.632; 22.20% | 62.625; 14.63% | 77.459; 23.69% |
| Total of C10BA* | 0 | 0 | 0 | 0 | 0 | 0 | 0 | 0 | 0 | 0 | 0.042 | 0.209; 392.20% |
| Total of C10BX* | 0.01 | 0.033; 215.10% | 0.024; -28.17% | 0.024; 0% | 0.021; -11.42% | 0.015; -27.20% | 0.331; 2071.33% | 0.452; 36.57% | 2.427; 436.56% | 7.428; 206.13% | 13.455; 81.13% | 18.394; 36.27% |
| Total of combination medicine* | 0.01 | 0.033; 215.10% | 0.024; -28.17% | 0.024; 0% | 0.021; -11.42% | 0.015; -27.20% | 0.331; 2071.33% | 0.452; 36.57% | 2.427; 436.56% | 7.428; 206.13% | 13.498; 81.70% | 18.602; 37.82% |
| Statins, total | 8.284 | 10.514; 26.92% | 12.974; 23.40% | 14.949; 15.22% | 19.516; 30.55% | 21.507; 10.20% | 29.613; 37.69% | 38.071; 28.56% | 47.133; 23.80% | 62.060; 31.67% | 76.122; 22.66% | 96.061; 26.19% |

DDD – defined daily doses, TID – per one thousand inhabitants per day, C10AA - HMG CoA reductase inhibitors, C10BA - Combinations of various lipid modifying agents, C10BX – Lipid modifying agents in combination with other drugs.

* DDD calculated according to the DDD of statin in combination
